# Prognostic ability of inflammation-based markers in radioembolization for hepatocellular carcinoma

**DOI:** 10.20517/2394-5079.2020.57

**Published:** 2020-10-12

**Authors:** Grant Yoneoka, Kliment Bozhilov, Linda L. Wong

**Affiliations:** 1 Transplant Center, The Queen’s Medical Center, Honolulu, HI 96813, USA; 2 Department of Surgery, University of Hawaii, John A. Burns School of Medicine, Honolulu, HI 96813, USA

**Keywords:** Neutrophil-to-lymphocyte ratio, platelet-to-lymphocyte ratio, transarterial radioembolization, hepatocellular carcinoma

## Abstract

**Aim:**

Inflammation-based markers, such as the neutrophil-to-lymphocyte ratio (NLR) and platelet-to-lymphocyte ratio (PLR), have recently been used as prognostic indicators in hepatocellular carcinoma (HCC). We aimed to determine whether NLR and PLR may predict response to yttrium-90 transarterial radioembolization (TARE) as primary treatment for HCC.

**Methods:**

We performed a retrospective review of a prospectively collected database of HCC cases (1994–2019) and selected patients who received TARE as primary treatment (*n* = 42). Laboratory studies were used to calculate NLR and PLR. Response to TARE was determined using the modified response evaluation criteria in solid tumors (mRECIST). Patients were classified as non-responders (stable or progressive disease) or responders (partial or complete response) to treatment based on mRECIST.

**Results:**

Receiver operating characteristic curves identified a pre-treatment NLR cutoff of ≥ 2.83 and a pre-treatment PLR cutoff of ≥ 83 for predicting non-response to treatment. Pre-treatment NLR ≥ 2.83 was the only significant predictor of non-response to TARE in multivariate logistic regression analysis (odds ratio 7.83, *P* = 0.036). On time to progression analysis, both pre-treatment NLR ≥ 2.83 and pre-treatment PLR ≥ 83 were associated with a higher proportion of tumor progression at 6 months post-treatment (43.6% *vs*. 10.0%, *P* = 0.014, log-rank) and (38.6% *vs*. 0%, *P* = 0.010, log-rank), respectively.

**Conclusion:**

NLR confers prognostic value and may be superior to PLR in determining response to TARE as primary treatment for HCC. Future studies are necessary to validate these findings in a larger cohort.

## INTRODUCTION

Hepatocellular carcinoma (HCC) is the most common primary liver cancer and the fourth most common cause of cancer-related death worldwide^[1]^. In the United States, the overall prognosis for HCC is poor, with a 5-year survival rate of 10%^[2]^. Generally accepted curative therapies include liver resection or transplantation. Unfortunately, patients with advanced disease are usually not amenable to surgical intervention. For patients with unresectable tumors, transarterial chemoembolization (TACE) or transarterial radioembolization (TARE) with yttrium-90 (Y90) can be considered to treat or downstage disease to qualify for curative surgery. Although TACE has been the mainstay of treatment for intermediate-stage tumors, TARE has a distinct advantage in that it can be used in portal venous thrombosis and has a better adverse effect profile with similar efficacy to TACE^[3–5]^.

Response to TARE is measured by the modified response evaluation criteria in solid tumors (mRECIST) using either contrast-enhanced computed tomography (CT) or magnetic resonance imaging (MRI)^[6]^. Depending on individual center protocols, initial images are performed 1 to 3 months post-treatment. Unfortunately, tumors that are non-responsive to TARE may progress while waiting for subsequent imaging. Therefore, prognostic biomarkers are needed to help predict which patients will benefit from TARE.

The serum marker alpha-fetoprotein (AFP), widely used as a screening tool for HCC, has been shown to have prognostic value in treatment^[7,8]^. However, AFP is also elevated in non-tumor environments and is not particularly sensitive for small tumors^[9]^. Liquid biopsy, which detects circulating tumor cells or nucleic acids, is a promising alternative to AFP but is not yet widely available^[10]^. Recently, inflammatory markers such as the neutrophil-to-lymphocyte ratio (NLR) and platelet-to-lymphocyte ratio (PLR) have gained popularity as prognostic indicators in cancer^[11–14]^. While the mechanism behind these markers is not precisely understood, a proinflammatory environment along with thrombocytosis has been associated with tumor growth and survival^[15–17]^.

Previous studies have highlighted the clinical utility of NLR and PLR as prognostic markers for HCC after liver resection, transplantation, and TACE^[12,14,18–20]^. However, the use of NLR in combination with PLR for TARE has not been well established. This study aimed to understand the prognostic value of NLR and PLR in patients who received TARE as a first-line therapy for HCC.

## METHODS

### Patients

This was a retrospective review from a prospectively collected database of 1,442 patients diagnosed with HCC from 1993 to 2019. All patients were referred to a group of hepatobiliary surgeons affiliated with a tertiary medical center in Hawaii that has the only liver center and liver transplant program in the state. This surgical group evaluates approximately 60%−70% of all the cases of HCC in Hawaii and includes referrals from the American territories of the Pacific Basin. We selected patients who received TARE as a primary treatment for HCC. Patients were excluded if they had a previous liver resection, liver transplant, any systemic therapy or locoregional therapy prior to TARE. Patients were also excluded if they received adjuvant therapy following TARE but prior to follow-up imaging. Patients who had initial follow-up later than 12 months were additionally excluded. We included patients who had two separate TARE treatments for bilateral or extensive disease. These Y90 treatments were typically done about 1 month apart, and imaging tests were done 3 months after treatment. This retrospective chart and imaging review study was approved by the Institutional Review Board at the Queen’s Medical Center and was determined to be exempt from needing informed consent.

The diagnosis of HCC was made with histologic confirmation of HCC with biopsy or with contrast-enhanced imaging (CT or MRI) which demonstrated liver mass or masses with LI-RADS 5 criteria, an arterial phase hyperenhancement and one or more of the following, “washout” on venous phase, an enhancing capsule or threshold growth. These criteria were also consistent with the Organ Procurement and Transplantation class 5 criteria.

Pre-treatment imaging was performed using either CT or MRI. Pre-treatment tumor size was defined as the sum of the diameters of all enhancing lesions. All images were taken within 6 months prior to TARE. All patients were evaluated by an interventional radiologist, hepatologist and surgeon and cases were discussed at a multidisciplinary hepatobiliary conference. Patients were not candidates for TARE if they had a total bilirubin above 2.0 mg/dL or evidence of extrahepatic spread of disease. A Y90 arterial mapping procedure was performed to identify the tumor(s), vascular branches supplying the tumor and degree of lung shunting with ^99m^Tc-macroaggregated albumin. Patients with greater than 10% lung shunting were not candidates for TARE.

Radioembolization was performed with Y90 delivered via glass microspheres (TheraSphere, Boston Scientific, USA) or resin microspheres (SIR-Spheres, Sirtex Medical, Australia). All procedures were performed by one of seven interventional radiologists who comprise the only group that performs complex hepatobiliary interventions in Hawaii.

Post-treatment imaging was performed at approximately 3-month and 6-month intervals. Response to TARE was determined using mRECIST. Patients with complete response (CR) or partial response (PR) according to mRECIST were further classified into a response group, while patients with stable disease (SD) or progressive disease (PD) were classified into a non-response group. For patients who received both a 3-month and 6-month scan, the 6-month scan was used to determine overall response to treatment.

### Data collected

Collected demographic information included patient age, sex and race. Medical history included height, weight, body mass index, diabetes mellitus, hyperlipidemia, hypertension, infection with hepatitis B or hepatitis C, significant alcohol use (> 2 alcoholic beverages daily for 10 years), smoking history, non-alcoholic fatty liver disease, non-alcoholic steatohepatitis, ascites, encephalopathy, cirrhosis, AFP and normal AFP (< 20 ng/mL).

Laboratory studies included prothrombin time, international normalized ratio, creatinine, aspartate transaminase, alanine aminotransferase, bilirubin, albumin, white blood cell count, neutrophil count, lymphocyte count and platelet count. Laboratory values were obtained prior to TARE and approximately 2 weeks, 3 months and 6 months post-treatment. NLR was defined as the ratio between the absolute neutrophil count and the absolute lymphocyte count. PLR was defined as the ratio between the absolute platelet count and the absolute lymphocyte count. Date of laboratory draws were used to determine temporal trends in NLR and PLR following treatment. Albumin-bilirubin (ALBI) grade and Child-Pugh class were calculated from baseline laboratory values.

After identifying patients and collecting baseline data from the prospectively collected database, individual charts were queried to obtain detailed imaging reports. Imaging was reviewed and measured retrospectively by a single physician. Collected imaging data included pre-treatment tumor size, post-treatment tumor size, mRECIST, and dates of imaging and treatment.

### Statistical analysis

Receiver operator characteristic (ROC) curves were used to determine optimal NLR and PLR cutoffs. Cutoff points were selected by maximizing Youden’s index. Mean comparisons were analyzed using Welch’s *t*-test. Categorical comparisons were performed using Fisher’s exact test. Independent predictors of response to treatment were determined using univariate logistic regression. Variables that were significant in the univariate analysis were included in the multivariate logistic regression model. Time to progression (TTP) was defined as the date of treatment until the date of PD based on mRECIST. Patients who did not reach the endpoint were censored based on their last imaging date. TTP was analyzed via the Kaplan-Meier method and compared using the log-rank test. All tests were two-tailed, and *P* < 0.05 was considered statistically significant. Statistical analyses were performed using SPSS version 26 (IBM, USA), Jamovi version 1.0.8 and GraphPad Prism8 (GraphPad Software, USA).

## RESULTS

### Cohort characteristics

In this cohort of 1,442 patients with HCC, a total of 276 TARE procedures were performed. Of those patients, 77 received TARE as primary treatment for HCC, and 42 patients met criteria for this study. Seven patients received a second TARE procedure within a month of the first: six were for bilateral disease, and one patient had extensive disease that was completed in 2 separate sessions to treat the entire lobe. The characteristics of this cohort are described in Table 1. The mean age of the cohort was 66.8 years [standard deviation (s.d.) 11.3 years]. There were 30 males and 12 females. Asian represented the largest ethnicity (61.9%), followed by Caucasian (19.0%), Pacific Islander (14.3%) and Hispanic (4.8%). There were 19 ALBI grade 1 patients, 20 ALBI grade 2 patients and 3 ALBI grade 3 patients. There were 33 Child-Pugh class A patients and 9 Child-Pugh class B patients. There were no Child-Pugh class C patients. The mean pre-treatment AFP was 2,023 ng/mL (s.d. 7605 ng/mL). Twenty-three patients had normal AFP prior to TARE. The mean total tumor size was 7.0 cm (s.d. 4.0 cm), and the mean number of tumors was 1.71 (s.d. 1.07).

### Determination of cutoff points and comparison between groups

**Figure 1.**
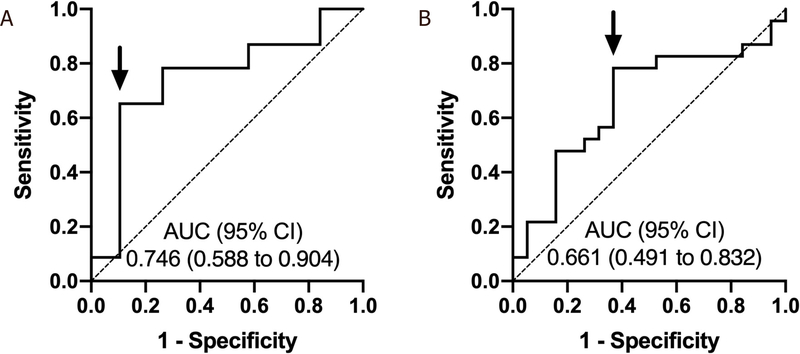
Receiver operating characteristic curves for pre-treatment NLR (A) and PLR (B) in predicting non-response to TARE. The cutoff points for pre-treatment NLR and PLR were 2.83 and 83, respectively. Arrows depict selected cutoff points. NLR: neutrophil-to-lymphocyte ratio; PLR: platelet-to-lymphocyte ratio; AUC: area under the curve; CI: confidence interval; TARE: transarterial radioembolization

ROC analysis identified a pre-treatment NLR cutoff of 2.83 [area under the curve (AUC) = 0.746, 95% confidence interval (CI): 0.588–0.904, sensitivity: 65.2% and specificity: 89.5%] [Figure 1A] and a pre-treatment PLR cutoff of 83 (AUC = 0.661, 95%CI: 0.491–0.832, sensitivity: 78.3% and specificity: 63.2%) for predicting non-response to TARE [Figure 1B].

The mean age was higher in the pre-treatment NLR ≥ 2.83 group than the pre-treatment NLR < 2.83 group (72.2 *vs.* 63.1, *P* = 0.008) [Table 2]. Pre-treatment NLR ≥ 2.83 was associated with ALBI grade ≥ 2 (*P* = 0.029). The pre-treatment NLR ≥ 2.83 group had a higher mean neutrophil count (3.97 × 10^9^/L *vs.* 2.51 × 10^9^/L, *P* = 0.001) but lower mean lymphocyte count (0.98 × 10^9^/L *vs.* 1.71 × 10^9^/L, *P* = 0.001) compared to the pre-treatment NLR < 2.83 group.

The mean age was higher in the pre-treatment PLR ≥ 83 group than the pre-treatment PLR < 83 group (72.1 *vs.* 59.0, *P* = 0.001) [Table 3]. Pre-treatment PLR ≥ 83 was associated with hyperlipidemia (*P* = 0.004) and Child-Pugh class B (*P* = 0.006). The pre-treatment PLR ≥ 83 group had a higher mean platelet count (186.2 × 10^9^/L *vs.* 97.5 × 10^9^/L, *P* = 0.001) but lower mean lymphocyte count (1.24 × 10^9^/L *vs.* 1.67 × 10^9^/L, *P* = 0.048) compared to the pre-treatment PLR < 83 group.

### Response to treatment

**Figure 2.**
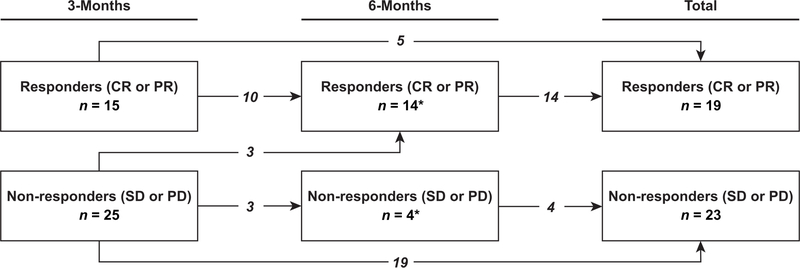
Changes in response to transarterial radioembolization over time. Response was defined as complete response (CR) or partial response (PR) using modified response evaluation criteria in solid tumors. Non-response was defined as stable disease (SD) or progressive disease (PD). Arrows depict changes in response between 3-month and 6-month imaging. The total box represents the overall count of responders and non-responders to treatment. Two patients (one responder and one non-responder) did not receive 3-month imaging, and initial response was evaluated at 6-month follow-up instead (asterisk)

The change in response to TARE over time is shown in Figure 2. There were 15 responders to treatment (4 CR, 11 PR) and 25 non-responders to treatment (18 SD, 7 PD) at 3-month follow-up. At 6-month follow-up, there were 14 responders to treatment (6 CR, 8 PR) and 4 non-responders to treatment (4 SD). In total, using the latest available scan to determine overall response, there were 19 responders to treatment (7 CR, 12 PR) and 23 non-responders to treatment (16 SD, 7 PD). Of the causes of progression in the 7 patients with PD, 1 had new intrahepatic lesions, 4 had an increase in size of existing intrahepatic lesion(s) and 2 had both an increase in size of an existing intrahepatic lesion and a new intrahepatic lesion.

### NLR and PLR for non-responders and responders

**Figure 3.**
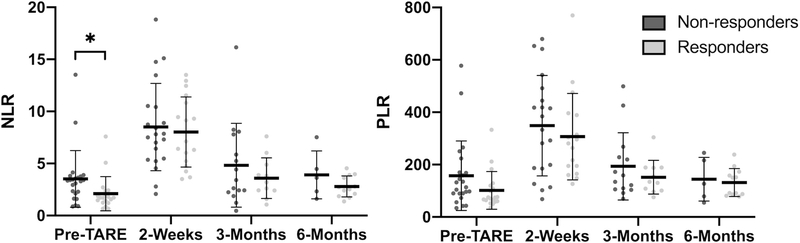
Mean NLR and PLR for non-responders and responders to TARE. The mean pre-treatment NLR was higher for non-responders than for responders (3.5 *vs*. 2.1, *P* = 0.045) (asterisk). Error bars represent the standard deviation. NLR: neutrophil-to-lymphocyte ratio; PLR: platelet-to-lymphocyte ratio; TARE: transarterial radioembolization

The mean values of NLR and PLR at pre-treatment, 2 weeks post-treatment, 3 months post-treatment and 6 months post-treatment are shown in Figure 3. The mean pre-treatment NLR for non-responders was ignificantly higher than that for responders (3.5 *vs.* 2.1, *P* = 0.045). There were no statistically significant differences in PLR or NLR for other time points.

### Predictors of response to TARE

Predictors of response to treatment are shown in Table 4. Univariate predictors of non-response to TARE included age ≥ 65 [odds ratio (OR) = 4.06, 95%CI: 1.12–14.80, *P* = 0.034], ALBI grade ≥ 2 (OR = 6.14, 95%CI: 1.60–23.50, *P* = 0.008), pre-treatment NLR ≥ 2.83 (OR = 15.94, 95%CI: 2.92–87.06, *P* = 0.001) and pre-treatment PLR ≥ 83 (OR = 6.17, 95%CI: 1.58–24.05, *P* = 0.009). On multivariate analysis, pre-treatment NLR ≥ 2.83 was a significant variable associated with non-response to TARE (OR = 7.83, 95%CI: 1.14–53.61, *P* = 0.036), while pre-treatment PLR ≥ 83 was not a significant variable associated with non-response to TARE (OR = 3.01, 95%CI: 0.49–18.34, *P* = 0.232).

### Time to progression

**Figure 4.**
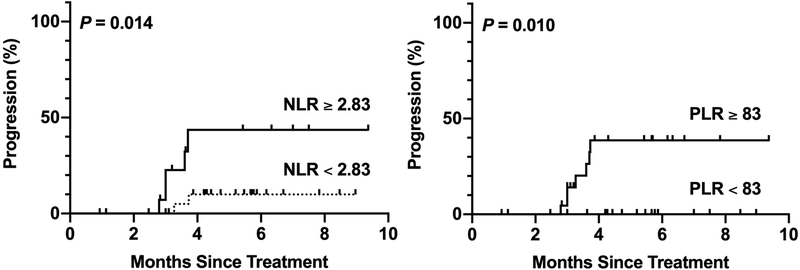
Kaplan-Meier curves for time to progression grouped according to pre-treatment NLR and pre-treatment PLR cutoff values. Censored events are represented by vertical lines. NLR: neutrophil-to-lymphocyte ratio; PLR: platelet-to-lymphocyte ratio

TTP for pre-treatment NLR and pre-treatment PLR is shown in Figure 4. Pre-treatment NLR ≥ 2.83 was associated with a higher proportion of tumor progression than pre-treatment NLR < 2.83 at 6 months post-TARE (43.6% *vs.* 10.0%, *P* = 0.014, log-rank). Pre-treatment PLR ≥ 83 was also associated with a higher proportion of tumor progression than pre-treatment PLR > 83 at 6 months post-TARE (38.6% *vs.* 0%, *P* = 0.010, log-rank). Median TTP was not reached in any group.

## DISCUSSION

Traditional ways of monitoring response to TARE have relied on imaging techniques such as CT or MRI. While imaging has been the best modality to demonstrate changes in tumor size, it may require months to see a visible response. Patients who did not respond to therapy during this time may have had disease progression. Therefore, it would be advantageous to find prognostic markers that can predict tumor response or progression prior to subsequent imaging. Inflammation-based markers, such as NLR and PLR, may provide an ideal solution as they are relatively easy to obtain from routine laboratory results and have established prognostic value in previous studies on HCC^[11–14]^.

This study sought to determine the ability of NLR and PLR to predict response to TARE as primary treatment for HCC. We demonstrated that a pre-treatment NLR ≥ 2.83 was associated with non-response to TARE in both univariate and multivariate analysis. These findings were in agreement with Taussig *et al.*^[21]^, who previously used a similar grouping system based on mRECIST to demonstrate that an elevated NLR is associated with tumor progression after intra-arterial therapy. Although other studies have shown that an elevated NLR was associated with poor overall survival following TARE, none of these studies reported specifically on tumor progression based on imaging^[22,23]^. These results taken together suggest that NLR may be a valuable prognostic marker in TARE.

Notably, we found that an elevated pre-treatment PLR predicted non-response to TARE in univariate analysis but was not a significant variable in our multivariate model. This suggests that the pre-treatment NLR may be superior to pre-treatment PLR in predicting non-response to TARE. Nonetheless, this result may be limited by our small sample size. To our knowledge, this was the first study to examine the prognostic capabilities of PLR in TARE based specifically on tumor response to therapy. D’emic *et al.*^[24]^ previously suggested in their study of 116 patients who received selective internal radiation therapy that pre-treatment PLR > 78 was the most predictive serum marker associated with improved overall survival. However, it is difficult to make definitive conclusions about NLR and PLR in HCC as their study also included other cancer types and only 37 patients had HCC. Future studies are therefore needed to compare the prognostic capabilities of PLR compared to NLR in TARE.

On TTP analysis, we found that pre-treatment NLR ≥ 2.83 and pre-treatment PLR ≥ 83 were both associated with a higher proportion of tumor progression at 6 months post-TARE. The median TTP was not yet reached in all groups. This is consistent with previous results published by Salem *et al.*^[3]^, who found that the median TTP for radioembolization was greater than 26 months. On the basis of these results, both the pre-treatment NLR and pre-treatment PLR may have utility in predicting tumor progression at 6 months following TARE. Nonetheless, the prognostic value of NLR could have a distinct advantage over PLR because pre-treatment NLR < 2.83 was also associated with response to treatment in our multivariate logistic regression analysis. NLR may therefore have greater clinical utility than PLR as pre-treatment NLR was predictive of both tumor progression and potential response to therapy in our cohort. In comparison, pre-treatment PLR was only predictive of tumor progression in our TTP analysis.

The ALBI grade was a newer model proposed by Johnson *et al.*^[25]^ that offered better discriminatory capabilities compared to the Child-Pugh class. While other studies reported that the ALBI grade was predictive of survival following TARE, our multivariate model did not find the ALBI grade helpful in predicting response to TARE^[26,27]^. Since the ALBI grade likely reflects underlying liver function, it may be more suitable for determining longer-term overall survival following TARE, rather than predicting specific tumor response to treatment.

The underlying mechanism behind NLR and PLR is not well understood. However, it is generally recognized that inflammation plays a key role in the development of cancer^[15,17]^. Neutrophils can favor a pro-mutagenic state with the abundant release of reactive oxygen species and proteases^[28]^. In addition, platelets may support a pro-tumor microenvironment with the release of angiogenic factors such as vascular endothelial growth factor and basic fibroblastic growth factor^[29]^. These observations, coupled with the fact that lymphopenia has been associated with advanced disease in various tumors, may be reflected in systemic inflammation-based markers such as NLR and PLR^[30]^. Nonetheless, more research is needed to better understand the basis of these two markers.

This study was limited in that this was a single center study with a small sample size. This study was also retrospective, and the exact timings of imaging and laboratory studies were not collected consistently as part of a study protocol. Missing data in some patients may have also contributed to our small sample size. In addition, several patients may have had laboratory data collected for other medical issues unrelated to TARE, which may have influenced NLR and PLR.

Despite these limitations, the results of this study suggest that the pre-treatment NLR may predict response to TARE as primary treatment for HCC. Furthermore, the pre-treatment NLR may also have better prognostic value than the pre-treatment PLR or ALBI grade in predicting tumor response to therapy. These findings may help clinicians identify patients who are expected to respond poorly to TARE prior to treatment and enable them to consider additional or alternative therapies. However, future studies that examine NLR and PLR in a larger cohort prospectively will be necessary to draw definitive conclusions about the prognostic capabilities of these two inflammation-based markers.

## Figures and Tables

**Table 1.** Cohort characteristics

Characteristic	
Mean age in years (s.d.)	66.8 (11.3)
Males	30 (71.4%)
Ethnicity	
Caucasian	8 (19.0%)
Pacific Islander	6 (14.3%)
Asian	26 (61.9%)
Hispanic	2 (4.8%)
Hepatitis B surface Ag positive	5 (11.9%)
Hepatitis B core Ab positive	6 (14.3%)
Hepatitis C positive	19 (45.2%)
Alcohol abuse	14 (33.3%)
NASH/NAFLD	13 (31.0%)
Mean BMI (s.d.)	26.7 (4.9)
BMI 30 or higher	8 (19.0%)
Smoking history	27 (64.3%)
Diabetes mellitus	17 (40.5%)
Hyperlipidemia	19 (45.2%)
Hypertension	32 (76.2%)
ALBI	
Grade 1	19 (45.2%)
Grade 2	20 (47.6%)
Grade 3	3 (7.1%)
Child-Pugh class	
A	33 (78.6%)
B	9 (21.4%)
Mean AFP in ng/mL (s.d.)	2023 (7605)
Normal AFP	24 (57.1%)
Mean total tumor size in cm (s.d.)	7.0 (4.0)
Number of tumors (s.d.)	1.71 (1.07)

s.d.: standard deviation; NASH: non-alcoholic steatohepatitis; NAFLD: non-alcoholic fatty liver disease; BMI: body mass index; ALBI: albumin-bilirubin; AFP: alpha-fetoprotein

**Table 2.** Comparison of pre-treatment NLR < 2.83 and NLR ≥ 2.83 groups

	Pre-treatment NLR < 2.83 *(n* = 25)	Pre-treatment NLR ≥ 2.83 *(n* = 17)	*P*-value
Mean age in years (s.d.)	63.1 (11.0)	72.2 (9.7)	0.008
Male sex	18 (72.0%)	12 (70.6%)	1.000
Hepatitis B	5 (20.0%)	0 (0%)	0.070
Hepatitis C	14 (56.0%)	5 (29.4%)	0.120
Alcohol abuse	8 (32.0%)	6 (35.3%)	1.000
NASH/NAFLD	5 (20.0%)	8 (47.1%)	0.092
Mean BMI (s.d.)	26.2 (4.4)	27.4 (5.5)	0.448
Smoking history	14 (56.0%)	13 (76.5%)	0.207
Diabetes mellitus	8 (32.0%)	9 (52.9%)	0.212
Hyperlipidemia	8 (32.0%)	11 (64.7%)	0.059
Hypertension	18 (72.0%)	14 (82.4%)	0.490
ALBI grade ≥ 2	10 (40.0%)	13 (76.5%)	0.029
Child-Pugh class B	4 (16.0%)	5 (29.4%)	0.446
Mean neutrophils (10^9^/L) (s.d.)	2.51 (0.98)	3.97 (1.18)	0.001
Mean lymphocytes (10^9^/L) (s.d.)	1.71 (0.66)	0.98 (0.42)	0.001
Mean AFP in ng/mL (s.d.)	2157 (8765)	1826 (5734)	0.883
Normal AFP	15 (60.0%)	9 (52.9%)	0.755
Mean total tumor size in cm (s.d.)	6.1 (3.6)	8.3 (4.3)	0.088
Number of tumors (s.d.)	1.76 (1.01)	1.65 (1.17)	0.748

NLR: neutrophil-to-lymphocyte ratio; s.d.: standard deviation; NASH: non-alcoholic steatohepatitis; NAFLD: non-alcoholic fatty liver disease; BMI: body mass index; ALBI: albumin-bilirubin; AFP: alpha-fetoprotein

**Table 3.** Comparison of pre-treatment PLR < 83 and PLR > 83 groups

	Pre-treatment PLR < 83 *(n* = 17)	Pre-treatment PLR ≥ 83 *(n* = 25)	*P*-value
Mean age in years (s.d.)	59.0 (9.1)	72.1 (9.6)	0.001
Males	14 (82.4%)	16 (64.0%)	0.300
Hepatitis B	2 (11.8%)	3 (12.0%)	1.000
Hepatitis C	11 (64.7%)	8 (32.0%)	0.059
Alcohol abuse	5 (29.4%)	9 (36.0%)	0.747
NASH/NAFLD	3 (17.6%)	10 (40.0%)	0.179
Mean BMI (s.d.)	26.9 (4.4)	26.5 (5.3)	0.772
Smoking history	12 (70.6%)	15 (60.0%)	0.531
Diabetes mellitus	5 (29.4%)	12 (48.0%)	0.339
Hyperlipidemia	3 (17.6%)	16 (64.0%)	0.004
Hypertension	12 (70.6%)	20 (80.0%)	0.714
ALBI grade ≥ 2	7 (41.2%)	16 (64.0%)	0.209
Child-Pugh class B	0 (0.0%)	9 (36.0%)	0.006
Mean platelets (10^9^/L) (s.d.)	97.5 (51.1)	186.2 (75.2)	0.001
Mean lymphocytes (10^9^/L) (s.d.)	1.67 (0.68)	1.24 (0.62)	0.048
Mean AFP in ng/mL (s.d.)	516 (1163)	3048 (9757)	0.211
Normal AFP	10 (58.8%)	14 (56.0%)	1.000
Mean total tumor size in cm (s.d.)	6.3 (4.0)	7.5 (4.0)	0.359
Number of tumors (s.d.)	2.06 (1.09)	1.48 (1.00)	0.090

OPLR: platelet-to-lymphocyte ratio; s.d.: standard deviation; NASH: non-alcoholic steatohepatitis; NAFLD: non-alcoholic fatty liver disease; BMI: body mass index; ALBI: albumin-bilirubin; AFP: alpha-fetoprotein

**Table 4.** Predictors of non-response to TARE

	Univariate analysis		Multivariate analysis	
	OR (95%CI)	*P*-value	OR (95%CI)	*P*-value
	
Age > 65	4.06 (1.12–14.80)	0.034	1.45 (0.21–10.17)	0.709
Male sex	0.82 (0.21–3.16)	0.769		
Hepatitis B	0.17 (0.02–1.68)	0.130		
Hepatitis C	0.86 (0.25–2.90)	0.801		
Alcohol abuse	1.80 (0.48–6.74)	0.383		
NASH/NAFLD	1.49 (0.39–5.67)	0.556		
BMI≥30	1.48 (0.31–7.21)	0.626		
Smoking history	1.09 (0.31–3.88)	0.890		
Diabetes mellitus	1.32 (0.38–4.58)	0.663		
Hyperlipidemia	2.82 (0.79–10.04)	0.110		
Hypertension	2.19 (0.52–9.33)	0.288		
ALBI grade ≥ 2	6.14 (1.60–23.50)	0.008	4.15 (0.80–21.52)	0.090
Child-Pugh class B	1.04 (0.24–4.59)	0.957		
Normal AFP	0.42 (0.12–1.50)	0.183		
Total tumor size ≥ 10 cm	3.00 (0.53–17.02)	0.215		
Multiple tumors	0.59 (0.17–2.06)	0.410		
Pre-treatment NLR ≥ 2.83	15.94 (2.92–87.06)	0.001	7.83 (1.14–53.61)	0.036
Pre-treatment PLR ≥ 83	6.17 (1.58–24.05)	0.009	3.01 (0.49–18.34)	0.232

OR: odds ratio; CI: confidence interval; NASH: non-alcoholic steatohepatitis; NAFLD: non-alcoholic fatty liver disease; BMI: body mass index; ALBI: albumin-bilirubin; AFP: alpha-fetoprotein; NLR: neutrophil-to-lymphocyte ratio; PLR: platelet-to-lymphocyte ratio; TARE: transarterial radioembolization

## References

[1] Yang JD, Hainaut P, Gores GJ, Amadou A, Plymoth A, A global view of hepatocellular carcinoma: trends, risk, prevention and management. Nat Rev Gastroenterol Hepatol 2019;16:589–604. 31439937 10.1038/s41575-019-0186-y PMC6813818

[2] McGlynn KA, London WT. The global epidemiology of hepatocellular carcinoma: present and future. Clin Liver Dis 2011;15:223–43, vii-x. 21689610 10.1016/j.cld.2011.03.006 PMC4141529

[3] Salem R, Gordon AC, Mouli S, Hickey R, Kallini J, Y90 radioembolization significantly prolongs time to progression compared with chemoembolization in patients with hepatocellular carcinoma. Gastroenterology 2016;151:1155–63.e2. 27575820 10.1053/j.gastro.2016.08.029 PMC5124387

[4] Moreno-Luna LE, Yang JD, Sanchez W, Paz-Fumagalli R, Harnois DM, Efficacy and safety of transarterial radioembolization versus chemoembolization in patients with hepatocellular carcinoma. Cardiovasc Intervent Radiol 2013;36:714–23. 23093355 10.1007/s00270-012-0481-2 PMC3594060

[5] Carr BI, Kondragunta V, Buch SC, Branch RA. Therapeutic equivalence in survival for hepatic arterial chemoembolization and yttrium 90 microsphere treatments in unresectable hepatocellular carcinoma: a two-cohort study. Cancer 2010;116:1305–14. 20066715 10.1002/cncr.24884 PMC2829376

[6] Lencioni R, Llovet JM. Modified RECIST (mRECIST) assessment for hepatocellular carcinoma. Semin Liver Dis 2010;30:52–60. 20175033 10.1055/s-0030-1247132 PMC12268942

[7] Duvoux C, Roudot-Thoraval F, Decaens T, Pessione F, Badran H, ; Liver Transplantation French Study Group. Liver transplantation for hepatocellular carcinoma: a model including α-fetoprotein improves the performance of Milan criteria. Gastroenterology 2012;143:986–94.e3; quiz e14–5. 22750200 10.1053/j.gastro.2012.05.052

[8] Berry K, Ioannou GN. Serum alpha-fetoprotein level independently predicts posttransplant survival in patients with hepatocellular carcinoma. Liver Transpl 2013;19:634–45. 23536495 10.1002/lt.23652

[9] Tateishi R, Yoshida H, Matsuyama Y, Mine N, Kondo Y, Diagnostic accuracy of tumor markers for hepatocellular carcinoma: a systematic review. Hepatol Int 2008;2:17–30. 19669276 10.1007/s12072-007-9038-x PMC2716871

[10] Li J, Han X, Yu X, Xu Z, Yang G, Clinical applications of liquid biopsy as prognostic and predictive biomarkers in hepatocellular carcinoma: circulating tumor cells and circulating tumor DNA. J Exp Clin Cancer Res 2018;37:213. 30176913 10.1186/s13046-018-0893-1 PMC6122633

[11] Templeton AJ, McNamara MG, Šeruga B, Vera-Badillo FE, Aneja P, Prognostic role of neutrophil-to-lymphocyte ratio in solid tumors: a systematic review and meta-analysis. J Natl Cancer Inst 2014;106:dju124. 24875653 10.1093/jnci/dju124

[12] Qi X, Li J, Deng H, Li H, Su C, Neutrophil-to-lymphocyte ratio for the prognostic assessment of hepatocellular carcinoma: a systematic review and meta-analysis of observational studies. Oncotarget 2016;7:45283–301. 27304193 10.18632/oncotarget.9942 PMC5216723

[13] Templeton AJ, Ace O, McNamara MG, Al-Mubarak M, Vera-Badillo FE, Prognostic role of platelet to lymphocyte ratio in solid tumors: a systematic review and meta-analysis. Cancer Epidemiol Biomarkers Prev 2014;23:1204–12. 24793958 10.1158/1055-9965.EPI-14-0146

[14] Li C, Wen TF, Yan LN, Li B, Wang WT, Postoperative neutrophil-to-lymphocyte ratio plus platelet-to-lymphocyte ratio predicts the outcomes of hepatocellular carcinoma. J Surg Res 2015;198:73–9. 26022997 10.1016/j.jss.2015.05.003

[15] Grivennikov SI, Greten FR, Karin M. Immunity, inflammation, and cancer. Cell 2010;140:883–99. 20303878 10.1016/j.cell.2010.01.025 PMC2866629

[16] Buergy D, Wenz F, Groden C, Brockmann MA. Tumor-platelet interaction in solid tumors. Int J Cancer 2012;130:2747–60. 22261860 10.1002/ijc.27441

[17] Greten FR, Grivennikov SI. Inflammation and Cancer: Triggers, mechanisms, and consequences. Immunity 2019;51:27–41. 31315034 10.1016/j.immuni.2019.06.025 PMC6831096

[18] Motomura T, Shirabe K, Mano Y, Muto J, Toshima T, Neutrophil-lymphocyte ratio reflects hepatocellular carcinoma recurrence after liver transplantation via inflammatory microenvironment. J Hepatol 2013;58:58–64. 22925812 10.1016/j.jhep.2012.08.017

[19] Pinato DJ, Sharma R. An inflammation-based prognostic index predicts survival advantage after transarterial chemoembolization in hepatocellular carcinoma. Transl Res 2012;160:146–52. 22677364 10.1016/j.trsl.2012.01.011

[20] Tian XC, Liu XL, Zeng FR, Chen Z, Wu DH. Platelet-to-lymphocyte ratio acts as an independent risk factor for patients with hepatitis B virus-related hepatocellular carcinoma who received transarterial chemoembolization. Eur Rev Med Pharmacol Sci 2016;20:2302–9. 27338055

[21] Taussig MD, Irene Koran ME, Mouli SK, Ahmad A, Geevarghese S, Neutrophil to lymphocyte ratio predicts disease progression following intra-arterial therapy of hepatocellular carcinoma. HPB (Oxford) 2017;19:458–64. 28190710 10.1016/j.hpb.2017.01.013

[22] Soydal C, Araz M, Nak D, Akkus P, Baltacioğlu MH, Analysis of prognostic factors in patients receiving transarterial radioembolization for unresectable hepatocellular carcinoma. Nucl Med Commun 2020;41:73–7. 31800509 10.1097/MNM.0000000000001122

[23] Sukato DC, Tohme S, Chalhoub D, Han K, Zajko A, The prognostic role of neutrophil-to-lymphocyte ratio in patients with unresectable hepatocellular carcinoma treated with radioembolization. J Vasc Interv Radiol 2015;26:816–24.e1. 25824315 10.1016/j.jvir.2015.01.038

[24] D’Emic N, Engelman A, Molitoris J, Hanlon A, Sharma NK, Prognostic significance of neutrophil-lymphocyte ratio and platelet-lymphocyte ratio in patients treated with selective internal radiation therapy. J Gastrointest Oncol 2016;7:269–77. 27034796 10.3978/j.issn.2078-6891.2015.108 PMC4783753

[25] Johnson PJ, Berhane S, Kagebayashi C, Satomura S, Teng M, Assessment of liver function in patients with hepatocellular carcinoma: a new evidence-based approach-the ALBI grade. J Clin Oncol 2015;33:550–8. 25512453 10.1200/JCO.2014.57.9151 PMC4322258

[26] Gui B, Weiner AA, Nosher J, Lu SE, Foltz GM, Assessment of the albumin-bilirubin (ALBI) grade as a prognostic indicator for hepatocellular carcinoma patients treated with radioembolization. Am J Clin Oncol 2018;41:861–6. 28418940 10.1097/COC.0000000000000384 PMC5645222

[27] Mohammadi H, Abuodeh Y, Jin W, Frakes J, Friedman M, Using the albumin-bilirubin (ALBI) grade as a prognostic marker for radioembolization of hepatocellular carcinoma. J Gastrointest Oncol 2018;9:840–6. 30505583 10.21037/jgo.2018.05.14 PMC6219966

[28] Coffelt SB, Wellenstein MD, de Visser KE. Neutrophils in cancer: neutral no more. Nat Rev Cancer 2016;16:431–46. 27282249 10.1038/nrc.2016.52

[29] Yan M, Jurasz P. The role of platelets in the tumor microenvironment: from solid tumors to leukemia. Biochim Biophys Acta 2016;1863:392–400. 26193075 10.1016/j.bbamcr.2015.07.008

[30] Ménétrier-Caux C, Ray-Coquard I, Blay JY, Caux C. Lymphopenia in cancer patients and its effects on response to immunotherapy: an opportunity for combination with cytokines? J Immunother Cancer 2019;7:85. 30922400 10.1186/s40425-019-0549-5 PMC6437964

